# The Integrative and Conjugative Element ICE*Pmi*W2 in *Proteus mirabilis* W2 Facilitates the Dissemination of Antibiotic‐Resistance Genes

**DOI:** 10.1155/cjid/5112699

**Published:** 2026-07-12

**Authors:** Yahan Cao, Wenchao Yu

**Affiliations:** ^1^ Yantai Institute of China Agricultural University, Yantai, 264670, China

**Keywords:** antibiotic resistance, aquaculture, horizontal gene transfer, integrative and conjugative element, *Proteus mirabilis*

## Abstract

**Background:**

The extensive use of antibiotics for treating infectious diseases leads to their release into the environment, which in turn results in antibiotic pollution and thereby facilitates the dissemination of antibiotic‐resistance genes (ARGs). Recently, despite the implementation of strict antibiotic usage restrictions, the accumulation of ARGs and multidrug‐resistant bacteria in the aquaculture environment continues to show a trend of persistent spread.

**Methods:**

The W2 strain was isolated in the presence of 32 μg/mL doxycycline. A broth microdilution assay was employed to determine the minimum inhibitory concentrations. Whole‐genome sequencing was conducted to characterize ARGs and their mobility through bioinformatics analysis. The spread of ARGs was detected by conjugation assays.

**Results:**

W2 strain was isolated from the wastewater of a crucian carp aquaculture plant in Jinan, China, and identified as *Proteus mirabilis*. W2 genome contained a 190,320 bp antibiotic‐resistance‐conferring integrative and conjugative element (ICE), named ICE*Pmi*W2. ICE*Pmi*W2 contains 21 ARGs, 14 conjugative transposon protein‐encoding genes, and one complex type I integron. No transconjugants were obtained using W2 as the donor strain and *Escherichia coli* 25DN as the recipient strain. However, evolutionary analysis revealed that ICE*Pmi*W2 likely evolved from ICEs of other *P. mirabilis* strains.

**Conclusions:**

The multiple‐antibiotic‐resistant *P*. *mirabilis* W2 strain with potential pathogenicity to aquatic animals was isolated, and the antibiotic‐resistance‐conferring ICE*Pmi*W2 was identified in *P*. *mirabilis* W2. Our findings suggest that ICE*Pmi*W2 of *P*. *mirabilis* W2 can potentially spread ARGs among environmental *P*. *mirabilis* strains.

## 1. Introduction

The spread of antibiotic resistance in aquaculture poses a severe threat to global public health, including an increased risk of treatment failures due to resistant infections in humans, the potential for zoonotic transmission of resistant bacteria from aquatic animals to humans via the food supply chain, and the dissemination of antibiotic resistance genes (ARGs) from aquaculture environments to natural water bodies, which can further accelerate the emergence of multidrug‐resistant (MDR) pathogens [1, 2]. Multiple‐antibiotic‐resistant *Vibrio* spp. and *Enterococcus* spp. were detected in retail shrimp in Northern California [3], and multiple‐antibiotic‐resistant strains of *Microbacterium* [4], *Gammaproteobacteria* [5], and *Shigella flexneri* [6] were also observed in aquatic environments.

Recently, increasing evidence has revealed the widespread occurrence of MDR *Proteus mirabilis* in aquaculture environments worldwide. In Pakistan, *P. mirabilis* carrying ESBL genes (e.g., *b* *l* *a*_CTX−M_ and *b* *l* *a*_OXA_) has been frequently isolated from fish and related environments [7]. In India, *P*. *mirabilis* strains isolated from sewage‐fed aquaculture systems exhibited strong biofilm‐forming capacity [8], while Indonesian studies reported high resistance rates to β‐lactams in *P. mirabilis* from African catfish [9]. In Nigeria, isolates from freshwater fish showed 100% resistance to amoxicillin [10]. In China, a survey of retail aquatic products revealed that seven *P. mirabilis* isolates were MDR, harboring clinically significant resistance genes including *b* *l* *a*_CTX−M_, *b* *l* *a*_NDM−1_, *fosA3*, and *tmexCD3-toprJ1* [11]. A recent genomic study further confirmed that *P. mirabilis* in China exhibits a significantly increasing resistance trend and serves as an important reservoir of ARGs under the One Health framework [12]. These findings underscore the urgent need to monitor and characterize *P. mirabilis* resistance in aquaculture settings.

*Proteus mirabilis* is widely present in soil and water environments, and it is also a type of normal intestinal flora in the human body. However, it can also cause severe infections in humans [13, 14]. Ninety percent of *Proteus* infections occur as a result of *P. mirabilis*, and these infections are considered to be community‐acquired [13]. *P*. *mirabilis* has been identified as an important zoonotic pathogen and is one of the main pathogens that cause urinary tract infections [15]; *P*. *mirabilis* can also cause wound infections, diarrhea, keratitis, gastroenteritis, and, in some cases, bacteremia [16–18].

Here, *P. mirabilis* W2 was isolated from the wastewater of a crucian carp aquaculture plant in Jinan, China. The aim of this study was to characterize the antibiotic resistance profile and the ICEs harbored by this strain, and to evaluate the potential role of ICE*Pmi*W2 in the dissemination of ARGs in aquaculture environments.

## 2. Materials and Methods

### 2.1. Isolation, Purification, and Identification of Strain W2

The W2 strain of *P*. *mirabilis* was isolated from a wastewater sample from a crucian carp aquaculture plant in Jinan, China. The water sample was diluted, plated onto Hektoen Enteric (HE) Agar medium (Oxoid, Britain) containing 32 mg/L doxycycline, and then incubated at 28°C for 24 h. The presumptive colony was subjected to three successive rounds of streaking onto fresh HE agar plates containing 32 mg/L doxycycline, and the resulting pure culture was designated as strain W2.

Molecular identification of strain W2 was performed as previously described [19]. 16S rRNA genes were amplified using the universal primers 27F (5′‐AGAGTTTGATCCTGGCTCAG‐3′) and 1492R (5′‐GGTTACCTTGTTACGACTT‐3′), and sequenced. Then, the sequences of 16S rRNA genes were aligned using BLAST (https://blast.ncbi.nlm.nih.gov/Blast.cgi) for preliminary identification.

### 2.2. Determination of the Minimum Inhibitory Concentrations (MICs)

To determine the MICs of different antibiotics for W2, the broth microdilution method was used as described previously [20], with *E*. *coli* ATCC 25922 serving as the quality control strain. To ensure reliability, each test was conducted with three biological replicates.

### 2.3. Whole‐Genome Sequencing, Annotation, and Analysis

W2 genome was sequenced by a Nanopore and BGISEQ‐500 platforms at BGI Co., Ltd. (Wuhan, China). Annotation of the W2 genome was performed using NCBI Prokaryotic Genome Annotation Pipeline (PGAP). Additional genome annotation was analyzed using RASTtk server [21, 22] and PATRIC [23]. Phylogenetic analysis of strain W2 was also analyzed using PATRIC. The whole‐genome average nucleotide identity (ANI) analysis was conducted using ANI calculator (https://www.ezbiocloud.net/tools/ani). ARGs were analyzed using ARDB [24], ARG‐ANNOT [25], and CARD [26]. Genomic islands were analyzed by IslandViewer 4 [27]. ISfinder and Integron Finder were used to analyze insertion sequence transposases and integrases [28]. Autonomous mobile elements in W2 genome were analyzed by ICEberg 2.0 [29].

### 2.4. Evolutionary Analysis of ICE*Pmi*W2

Evolutionary analysis of ICE*Pmi*W2 was analyzed using BLASTn (https://blast.ncbi.nlm.nih.gov/Blast.cgi) against the nonredundant (nr) nucleotide database. BLASTn searches against the nr database were performed under default parameters. Hits with ≥ 90% identity and ≥ 70% query coverage were retained. Synteny plots comparing ICE*Pmi*W2 with homologous ICEs were generated using Easyfig (version 2.2.5) as previously described [30].

### 2.5. Conjugation Experiments

Conjugation experiments were conducted to evaluate the horizontal transferability of ICE*Pmi*W2 from *P. mirabilis* W2 (donor) to *E. coli* 25DN (recipient) following a previously described protocol [31] with the following detailed steps. Strain 25DN was chosen as the recipient strain because it is resistant to sodium azide and capable of degrading X‐Gluc, which allows effective counter‐selection against the donor strain *P. mirabilis* W2 (sensitive to sodium azide and X‐Gluc‐negative). Moreover, 25DN is susceptible to doxycycline and chloramphenicol, facilitating the detection of transconjugants under selective pressure. Strains W2 and 25DN are, respectively, inhibited by doxycycline (or chloramphenicol) and sodium azide, and only the transconjugants can grow under pressure of doxycycline (or chloramphenicol) and degrade X‐Gluc to blue compounds. W2 and 25DN were mixed 1:1 and cultured on LB solid medium with sodium azide (1.7 mol/L), doxycycline/chloramphenicol (32 μg/mL), and X‐Gluc (5‐bromo‐4‐chloro‐3‐indolyl‐beta‐D‐glucuronic acid) to screen the transconjugants.

## 3. Results

### 3.1. Genomic Properties of *Proteus mirabilis* W2

**FIGURE 1:**
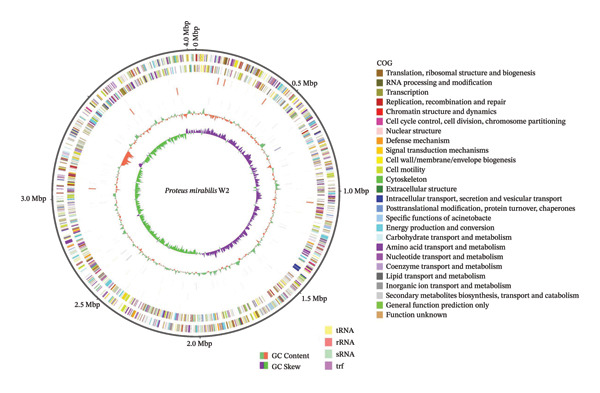
Comprehensive genomic analysis of *P. mirabilis* W2. From outer to inner rings: genome size and position markers; forward strand genes; reverse strand genes; forward strand ncRNA; reverse strand ncRNA; repeats; GC content; GC skew.

W2 was isolated from the Jinan (China) Freshwater Fish Farm. The 16S rDNA nucleotide sequence identity between W2 strain and *Proteus mirabilis* NCTC12441 strain is 99.94%, suggesting that W2 strain belongs to *Proteus* sp. W2 whole genome sequence was further sequenced and annotated (Figure 1). W2 carried a circular chromosome with a length of 4,037,899 base pairs, and the average GC content is 38.92%. No plasmid was identified in the W2 genome. W2 genome encodes 3543 proteins, 85 tRNA, 23 rRNA, and 23 sRNA. Based on the Clusters of Orthologous Groups (COG) functional classification, the predicted proteins were categorized into 26 functional groups. The most abundant categories were “Amino acid transport and metabolism” (E, 312 genes), “Carbohydrate transport and metabolism” (G, 289 genes), and “Transcription” (K, 274 genes).

**FIGURE 2:**
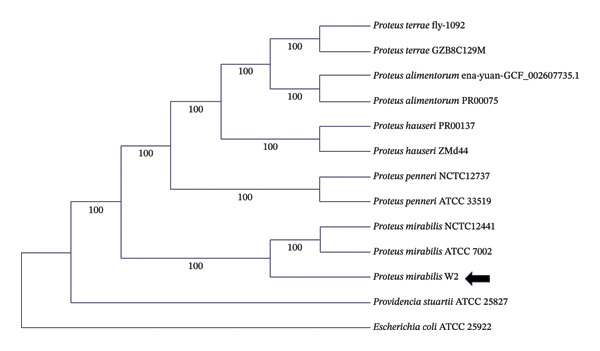
Phylogenetic tree of *P*. *mirabilis* W2 based on the genome sequence. Eleven strains from the genus *Proteus* and one strain from the genus *Providencia* were selected to analyze the phylogenetic relationship of W2. An *Escherichia coli* strain ATCC 25922 was used as the outgroup. The whole‐genome phylogenetic tree was constructed using the PATRIC server. W2 is most closely related to *P. mirabilis* NCTC12441 and *P. mirabilis* ATCC7002. The position of W2 is marked by a black arrow.

Phylogenetic tree analysis revealed that W2 formed a well‐supported clade with *P. mirabilis* NCTC12441 and *P. mirabilis* ATCC7002 (bootstrap value = 100%), indicating a close evolutionary relationship (Figure 2). ANI analysis revealed that the OrthoANIu value between the *P. mirabilis* W2 genome and *P. mirabilis* ATCC7002 genome was 99.05%, further confirming that W2 belongs to *P. mirabilis*. Based on both 16S rRNA gene sequence identity and whole‐genome phylogenetic analysis, strain W2 was unequivocally identified as *P. mirabilis*.

### 3.2. *Proteus* Mirabilis W2 Is a Multiantibiotic‐Resistant Strain

The antibiotic sensitivity test indicated that W2 exhibited high‐level resistance to all the tested antibiotics (Table 1), including tigecycline (32 μg/mL), doxycycline (> 128 μg/mL), ciprofloxacin (> 128 μg/mL), and enrofloxacin (> 128 μg/mL). Many ARGs in W2 genome were annotated (Table S1), including penicillin and cephalosporin resistance gene *b* *l* *a*_*O* *X* *A*−1_; tetracycline resistance genes *tet(J)* and *tet(C)*; beta‐lactam resistance genes *pbp3* and *b* *l* *a*_*C* *T* *X*−*M*−65_; macrolide ARGs *ereA* and *crp*; peptide ARGs *kpnH*, *arnT,* and *vanG*; and sulfonamide ARGs *sul1* and *sul2*. In addition, 147 virulence factor‐encoding genes were identified (Table S2), suggesting that W2 possesses the genetic potential for pathogenicity, though further experimental validation is required.

**TABLE 1:** Antibiotic susceptibility testing of *P. mirabilis* strain W2.

Antimicrobial agents	Antibiotic susceptibility, MIC (μg/mL)^a^
Tigecycline	32/R
Doxycycline	> 128/R
Ciprofloxacin	> 128/R
Enrofloxacin	> 128/R
Neomycin	> 128/R
Amikacin	64/R
Tylosin	> 128/R
Chloramphenicol	> 128/R
Florfenicol	> 128/R
Thiamphenicol	> 128/R
Flumequine	> 128/R
Sulfamethoxazole	32/R
Sulfadimidine	> 128/R
Sulfadiazine	> 128/R
Polymyxin E	16/R

^a^Bacterial antibiotic susceptibility was interpreted as per the CLSI guidelines. R, resistant.

### 3.3. Identification and Description of ICE*Pmi*W2

**FIGURE 3:**
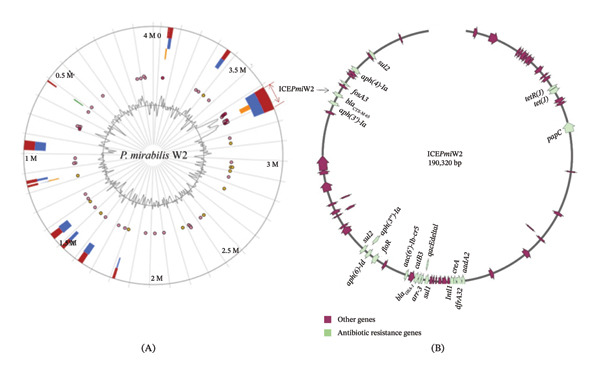
Genomic visualization of ICEP*mi*W2. (A) Genomic islands predicted in W2 genome. Red squares represent the combined results (integrated). (B) Arrangement of genes in ICEP*mi*W2. The green arrows indicate ARGs, and the dark purple arrows represent other genes.

IslandViewer 4 software analysis of W2 genome revealed that the region ranging from nucleotide positions 3,263,616 to 3,387,015 was a genomic island (GI). ICEberg 2.0 analysis further verified this GI as an ICE and this GI was named as ICE*Pmi*W2 (Figure 3). ICE*Pmi*W2 contains 190,320 bp and extends from position 3,198,373 to 3,388,692. ICE*Pmi*W2 is integrated into a hypothetical protein‐encoding gene (positions 3198042–3199907), and it is bounded by a 15‐bp direct repeat (DR) (5′‐ATATTATCAATAAGT‐3′). A total of 135 open reading frames were identified within ICE*Pmi*W2 (Table S3), including 21 ARGs and dozens of proteins involved in conjugation. ARGs in ICE*Pmi*W2 are shown in Figure 3B; these ARGs mediate resistance to chloramphenicol, tetracyclines, quinolones, aminoglycosides, and sulfonamides, consistent with the antibiotic resistance phenotype of the W2 strain. ICE*Pmi*W2 demonstrates a strong potential to accumulate ARGs, as evidenced by its carriage of a complex integron belonging to type I (Table 2).

**TABLE 2:** Features of the type I integron in ICEP*mi*W2.

Type	Annotation	Model^a^	Type^b^	Distance_2attC^c^
Type I integron (intI1)	protein	NA	complete	NA^∗^
	*attC*	attc_4	complete	NA
	ANT_3pp_I‐NCBIFAM	NF012157.0	complete	NA
	EreA‐NCBIFAM	NF000208.1	complete	NA
	*attC*	attc_4	complete	2164
	trim_DfrA1_like‐NCBIFAM	NF000330.1	complete	NA
	*intI*	intersection_tyr_intI	complete	NA
	SMR_qac_E‐NCBIFAM	NF000276.2	CALIN	NA
	*attC*	attc_4	CALIN	NA
	rifampin_ARR‐NCBIFAM	NF033144.1	CALIN	NA
	*attC*	attc_4	CALIN	490
	chloram_CatB‐NCBIFAM	NF000490.1	CALIN	NA
	*attC*	attc_4	CALIN	656
	*b* *l* *a*_OXA−1__like‐NCBIFAM	NF000388.2	CALIN	NA

^a^Model indicates the annotation source: “NFxxxxxx.x” denotes NCBIFAM protein family accession numbers. “attC_4” refers to the structural model used to identify *att*C recombination sites, whereas intersection_tyr_intI corresponds to the tyrosine recombinase family model of *intI*1.

^b^Type specifies whether the structure represents a complete integron (containing both intI and cassettes) or CALIN (cluster of *att*C sites lacking an integron integrase).

^c^Distance_2attC shows the nucleotide spacing between adjacent *att*C sites.

^∗^NA indicates data not applicable or unavailable.

### 3.4. Analysis of Horizontal Transfer Ability of ICEP*mi*W2

**FIGURE 4:**
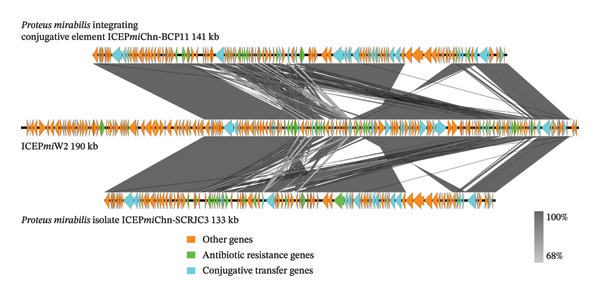
Synteny analysis of ICEP*mi*W2 with *Proteus mirabilis* integrating conjugative elements ICEP*mi*Chn‐BCP11 and ICEP*mi*Chn‐SCRJC3. Regions of high homology are shaded in gray. The blue arrows indicate conjugative transfer genes, the green arrows indicate ARGs, and the orange arrows represent other genes.

Homologous sequences of ICE*Pmi*W2 are present in the genomes of other *P. mirabilis* strains. ICE*Pmi*W2 exhibits high similarity to the *P*. *mirabilis* ICEs ICEP*mi*Chn‐BCP11 and ICEP*mi*Chn‐SCRJC3 (Figure 4). This observation suggests that ICE*Pmi*W2 likely evolved from ICEs in other *P. mirabilis* strains. A conjugation assay to evaluate the transfer of ICEP*mi*W2 from *P. mirabilis* W2 to *E. coli* 25DN was performed to test the mobility of ICEP*mi*W2 among Enterobacteriaceae species. However, no conjugant colonies were detected under the selective pressure of doxycycline (or chloramphenicol, each tested separately), indicating that ICEP*mi*W2 was not detected to be transferred to *E. coli* 25DN under the tested conditions.

## 4. Discussion

Aquaculture develops rapidly, with a global production of approximately 94 million tons and providing about 15% of animal protein in the human diet [32]. The aquaculture industry is mainly dominated by Asian countries, with China’s output in this field accounting for more than 60% [33]. Pathogens such as *Pseudomonas fluorescens*, *Yersinia ruckeri*, *Aeromonas hydrophila*, *Vibrio harveyi*, *Flavobacterium psychrophilum*, and *V. anguillarum* have been documented in aquaculture systems and can exert detrimental effects on the production of aquatic animals [1, 34]. An antibiotic‐resistant strain of *Proteus mirabilis* was reportedly isolated from an aquatic environment in Bangladesh [6]. Here, the multiple antibiotic‐resistant *P. mirabilis* strain W2 was isolated from a crucian carp aquaculture plant in China, indicating that attention should be paid to the potentially serious problems that this pathogen could cause in the aquaculture industry.

Of even greater concern, the potential for dissemination of antibiotic resistance via aquaculture pathogens poses a significant cross‐border public health risk. ICEs were reported to be the major way for spreading ARGs among bacteria [35, 36]. ICEs are defined as mobile genetic elements that integrate into the host genome and typically display mosaic and modular structures [37]. Capable of horizontal transfer via conjugation, ICEs encode a type IV secretion system and exist in an integrated state within the host genome. [38–40]. In this study, an antibiotic‐resistance‐conferring ICE, carrying 21 ARGs, was identified and named as ICEP*mi*W2 in the *P. mirabilis* W2 strain. Consistent with the two typical features of ICEs, ICEP*mi*W2 is inserted into the W2 genome, in a hypothetical protein‐encoding gene (positions 3198042–3199907), and encodes a type IV system, indicating ICEP*mi*W2 can disseminate ARGs via conjugation. ICE*Ci*POL15 in *Chryseobacterium indoltheticum* POL15 can disseminate ARGs to *E*. *coli* [41]. ICE*Csp*POL2 in *Chryseobacterium* sp. POL2, carries four types of ARGs, including carbapenem resistance gene, was found to transfer ARGs to *Elizabethkingia* sp*.* M6 strain but not to *E*. *coli* strains [35]. Our conjugation assays showed that no transconjugants were obtained using 25DN as the recipient strain, indicating that ICEP*mi*W2 may not disseminate ARGs to *E. coli*. Evolutionary analysis revealed that ICE*Pmi*W2 likely evolved from ICEP*mi*Chn‐BCP11 or ICEP*mi*Chn‐SCRJC3 of *P. mirabilis* strains, suggesting that ICE*Pmi*W2 in *P*. *mirabilis* W2 may potentially spread ARGs among environmental *P*. *mirabilis* strains.

## 5. Conclusions

*P*. *mirabilis* W2 was isolated from an aquaculture plant and sequenced. Genome annotation revealed that W2 encodes 147 virulence factors, suggesting that W2 is a potential pathogen for organisms. The 190‐kb antibiotic‐resistance‐conferring ICE*Pmi*W2 encodes 21 ARGs, which are associated with resistance to erythromycin, bleomycin, chloramphenicol, beta‐lactam antibiotics, tetracycline, fosfomycin, aminoglycosides, and sulfonamide. ICE*Pmi*W2 also encodes 14 conjugative transposon proteins and one complex type I integron. Conjugation assays showed that ICE*Pmi*W2 could not transfer ARGs to *E*. *coli* 25DN. However, evolutionary analysis revealed that ICE*Pmi*W2 likely evolved from ICEs of *P. mirabilis* strains, suggesting that ICE*Pmi*W2 in *P*. *mirabilis* W2 can potentially spread ARGs among environmental *P*. *mirabilis* strains. Given the importance of aquaculture in the global food industry, our findings suggest that monitoring for the presence of bacterial pathogens andARGs in aquaculture facilities is warranted.

## Author Contributions

Yahan Cao: investigation, methodology, formal analysis, validation, and writing–original draft. Wenchao Yu: project administration, supervision, and writing–review and editing.

## Funding

This work was supported by Research Initiation Project of Yantai Institute of China Agricultural University (No. 10110105).

## Conflicts of Interest

The authors declare no conflicts of interest.

## Supporting Information

Additional supporting information can be found online in the Supporting Information section.

## Supporting information


**Supporting Information** The following Supporting Information is available. Table S1 Annotated AMR genes in the genome of *Proteus mirabilis* W2. Table S2 Annotated VF genes in the genome of *Proteus mirabilis* W2. Table S3 Annotated genes in ICE*Pmi*W2.

## Data Availability

The W2 genome sequence has been deposited in GenBank (accession no. CP126338.1).
