# Supplementary material for: The Integrative and Conjugative Element ICEPmiW2 in Proteus mirabilis W2 Facilitates the Dissemination of Antibiotic‐Resistance Genes
Source: Can J Infect Dis Med Microbiol. 2026 Jul 12;2026:5112699. doi: 10.1155/cjid/5112699 (PMC13358208; doi:10.1155/cjid/5112699)
Supplement: Supplementary file 1 — Supporting Information The following Supporting Information is available. Table S1 Annotated AMR genes in the genome of Proteus mirabilis W2. Table S2 Annotated VF genes in the genome of Proteus mirabilis W2. Table S3 Annotated genes in ICEPmiW2. [file CJID-2026-5112699-s001.docx]

**Supplementary Material**

**The integrative and conjugative element ICE*Pmi*W2 in** ***Proteus mirabilis* W2 facilitates the dissemination of antibiotic-resistance genes**

Yahan Cao, Wenchao Yu*

Yantai Institute of China Agricultural University, Yantai 264670, China

*Address correspondence to Wenchao Yu, Yantai Institute of China Agricultural University, Yantai, China. E-mail: yuwenchao@cau.edu.cn.

**Table S1** Annotated AMR genes in the genome of *Proteus mirabilis* W2.

| start | end | strand | gene | Drug Class | Resistance Mechanism |
| --- | --- | --- | --- | --- | --- |
| 768179 | 771337 | - | *adeF* | fluoroquinolone antibiotic; tetracycline antibiotic | antibiotic efflux |
| 788157 | 788948 | - | *aadA* | aminoglycoside antibiotic | antibiotic inactivation |
| 788965 | 789438 | - | *dfrA1* | diaminopyrimidine antibiotic | antibiotic target replacement |
| 789435 | 790280 | - | *lnuF* | lincosamide antibiotic | antibiotic inactivation |
| 934487 | 936133 | + | *ArnT* | peptide antibiotic | antibiotic target alteration |
| 1035515 | 1035703 | + | *rsmA* | fluoroquinolone antibiotic; diaminopyrimidine antibiotic; phenicol antibiotic | antibiotic efflux |
| 1071721 | 1073238 | - | *KpnH* | macrolide antibiotic; fluoroquinolone antibiotic; aminoglycoside antibiotic; carbapenem; cephalosporin; penam; peptide antibiotic; penem | antibiotic efflux |
| 1400587 | 1401240 | - | *catA4* | phenicol antibiotic | antibiotic inactivation |
| 1727871 | 1729532 | + | *ArnT* | peptide antibiotic | antibiotic target alteration |
| 1945199 | 1946299 | + | *vanG* | glycopeptide antibiotic | antibiotic target alteration |
| 3225835 | 3227031 | + | *tet(J)* | tetracycline antibiotic | antibiotic efflux |
| 3288987 | 3289766 | - | *aadA2* | aminoglycoside antibiotic | antibiotic inactivation |
| 3289863 | 3291083 | - | *EreA* | macrolide antibiotic | antibiotic inactivation |
| 3291276 | 3291749 | - | *dfrA32* | diaminopyrimidine antibiotic | antibiotic target replacement |
| 3298327 | 3299166 | - | *sul1* | sulfonamide antibiotic | antibiotic target replacement |
| 3299160 | 3299507 | - | *qacEdelta1* | disinfecting agents and antiseptics | antibiotic efflux |
| 3299730 | 3300182 | - | *arr-3* | rifamycin antibiotic | antibiotic inactivation |
| 3300267 | 3300899 | - | *catB3* | phenicol antibiotic | antibiotic inactivation |
| 3301037 | 3301867 | - | *OXA-1* | carbapenem; cephalosporin; penam | antibiotic inactivation |
| 3303838 | 3304437 | - | *AAC(6')-Ib-cr6* | fluoroquinolone antibiotic; aminoglycoside antibiotic | antibiotic inactivation |
| 3312610 | 3313824 | + | *floR* | phenicol antibiotic | antibiotic efflux |
| 3314780 | 3315616 | - | *APH(6)-Id* | aminoglycoside antibiotic | antibiotic inactivation |
| 3315616 | 3316419 | - | *APH(3'')-Ib* | aminoglycoside antibiotic | antibiotic inactivation |
| 3316480 | 3317295 | - | *sul2* | sulfonamide antibiotic | antibiotic target replacement |
| 3352964 | 3354154 | - | *tet(C)* | tetracycline antibiotic | antibiotic efflux |
| 3356633 | 3357448 | - | *APH(3')-Ia* | aminoglycoside antibiotic | antibiotic inactivation |
| 3358973 | 3359848 | - | *CTX-M-65* | cephalosporin | antibiotic inactivation |
| 3362667 | 3363083 | + | *FosA3* | phosphonic acid antibiotic | antibiotic inactivation |
| 3365712 | 3366488 | + | *AAC(3)-IVa* | aminoglycoside antibiotic | antibiotic inactivation |
| 3366717 | 3367742 | + | *APH(4)-Ia* | aminoglycoside antibiotic | antibiotic inactivation |
| 3668883 | 3669515 | + | *CRP* | macrolide antibiotic; fluoroquinolone antibiotic; penam | antibiotic efflux |
| 3960172 | 3960963 | - | *aadA* | aminoglycoside antibiotic | antibiotic inactivation |
| 3960980 | 3961453 | - | *dfrA1* | diaminopyrimidine antibiotic | antibiotic target replacement |
| 3961450 | 3962295 | - | *lnuF* | lincosamide antibiotic | antibiotic inactivation |
| 3630 | 6044 | + | *gyrB* | fluoroquinolone antibiotic | antibiotic target alteration |
| 132827 | 134011 | + | *EF-Tu* | elfamycin antibiotic | antibiotic target alteration |
| 2840574 | 2842370 | - | *PBP3* | cephalosporin; cephamycin; penam | antibiotic target alteration |
| 3643007 | 3644191 | - | *EF-Tu* | elfamycin antibiotic | antibiotic target alteration |

**Table S2** Annotated VF genes in the genome of *Proteus mirabilis* W2.

| start | end | strand | gene | VFclass | Virulence factors |
| --- | --- | --- | --- | --- | --- |
| 2390570 | 2391352 | - | *flgG* | Adherence | Flagella |
| 2388643 | 2389749 | - | *flgI* | Adherence | Flagella |
| 2399074 | 2401164 | - | *flhA* | Adherence | Flagella |
| 2362660 | 2363382 | - | *fliA* | Adherence | Flagella |
| 2376963 | 2378336 | + | *fliI* | Adherence | Flagella |
| 2382775 | 2383545 | + | *fliP* | Adherence | Flagella |
| 191482 | 192744 | + |  | Adherence | LPS O-antigen (*P. aeruginosa*) |
| 190355 | 191485 | + |  | Adherence | LPS O-antigen (*P. aeruginosa*) |
| 2647244 | 2648581 | - | *pilR* | Adherence | Type IV pili biosynthesis |
| 3742634 | 3744055 | + | *pilR* | Adherence | Type IV pili biosynthesis |
| 991964 | 992983 | + | *pilT* | Adherence | Type IV pili biosynthesis |
| 3872102 | 3872725 | - | *ecpA* | Adherence | E. coli common pilus (ECP)(*Escherichia*) |
| 3016773 | 3019271 | - | *papC* | Adherence | P fimbriae(*Escherichia*) |
| 3016028 | 3016738 | - | *papD* | Adherence | P fimbriae(*Escherichia*) |
| 1582031 | 1582765 | + | *flmH* | Adherence | Polar flagella(*Aeromonas*) |
| 1390938 | 1391699 | + | *nueA* | Adherence | Polar flagella(*Aeromonas*) |
| 2224850 | 2225845 | + | *plr/gapA* | Adherence | Streptococcal plasmin receptor/GAPDH(*Streptococcus*) |
| 922363 | 922929 | - | *fimB* | Adherence | Type I fimbriae(*Escherichia*) |
| 319437 | 322058 | + | *fimD* | Adherence | Type I fimbriae(*Escherichia*) |
| 322062 | 322604 | + | *fimF* | Adherence | Type I fimbriae(*Escherichia*) |
| 322620 | 323132 | + | *fimG* | Adherence | Type I fimbriae(*Escherichia*) |
| 318701 | 319399 | + |  | Adherence | Type I fimbriae(*Klebsiella*) |
| 318034 | 318600 | + | *fimA* | Adherence | Type I fimbriae(*Klebsiella*) |
| 2673984 | 2674562 | - | *algU* | Antiphagocytosis | Alginate regulation |
| 3081941 | 3083293 | - | *mucP* | Antiphagocytosis | Alginate regulation |
| 192735 | 193808 | + | *rmlB* | Antiphagocytosis | Capsular polysaccharide(*Vibrio*) |
| 1310132 | 1311538 | + |  | Antiphagocytosis | Capsule(*Klebsiella*) |
| 56049 | 57215 | - | *ugd* | Antiphagocytosis | Capsule(*Klebsiella*) |
| 1016811 | 1018973 | - | *fpvA* | Iron uptake | Pyoverdine receptors |
| 1764883 | 1766145 | + | *hemA* | Iron uptake | Heme biosynthesis(*Haemophilus*) |
| 206206 | 207147 | - | *hemC* | Iron uptake | Heme biosynthesis(*Haemophilus*) |
| 862638 | 863924 | - | *hemL* | Iron uptake | Heme biosynthesis(*Haemophilus*) |
| 988097 | 989227 | - | *hemN* | Iron uptake | Heme biosynthesis(*Haemophilus*) |
| 3744085 | 3745458 | - | *hemN* | Iron uptake | Heme biosynthesis(*Haemophilus*) |
| 2136384 | 2138426 | + | *shuA* | Iron uptake | Heme transport(*Shigella*) |
| 2141343 | 2142137 | + | *shuV* | Iron uptake | Heme transport(*Shigella*) |
| 2138462 | 2139511 | + | *chuS* | Iron uptake | Heme uptake(*Escherichia*) |
| 2140342 | 2141346 | + | *chuU* | Iron uptake | Heme uptake(*Escherichia*) |
| 2723638 | 2725677 | - | *ireA* | Iron uptake | Iron-regulated element(*Escherichia*) |
| 1707282 | 1708190 | - | *sitA* | Iron uptake | Iron/manganese transport(*Escherichia*) |
| 1706398 | 1707285 | - | *sitB* | Iron uptake | Iron/manganese transport(*Escherichia*) |
| 1705484 | 1706395 | - | *sitC* | Iron uptake | Iron/manganese transport(*Escherichi*a) |
| 1704630 | 1705487 | - | *sitD* | Iron uptake | Iron/manganese transport(*Escherichia*) |
| 884949 | 885929 | + | *irp6B* | Iron uptake | Siderophore-dependent iron uptake system(*Corynebacterium*) |
| 941218 | 942693 | - | *aprA* | Protease | Alkaline protease |
| 1038692 | 1039207 | + | *luxS* | Quorum sensing | Autoinducer-2(*Vibrio*) |
| 1608512 | 1609186 | - | *phoP* | Regulation | PhoPQ(*Salmonella*) |
| 1607047 | 1608510 | - | *phoQ* | Regulation | PhoPQ(*Salmonella*) |
| 2477683 | 2478339 | + |  | Regulation | RcsAB(*Klebsiella*) |
| 1419708 | 1421066 | - |  | Secretion system | Hcp secretion island-1 encoded type VI secretion system (H-T11SS) |
| 1347458 | 1349752 | + | *clpV1* | Secretion system | Hcp secretion island-1 encoded type VI secretion system (H-T20SS) |
| 1409776 | 1413324 | + | *icmF1* | Secretion system | Hcp secretion island-1 encoded type VI secretion system (H-T23SS) |
| 3857427 | 3859598 | + | *vgrG1* | Secretion system | Hcp secretion island-1 encoded type VI secretion system (H-T26SS) |
| 1802190 | 1804280 | + | *vgrG1* | Secretion system | Hcp secretion island-1 encoded type VI secretion system (H-T26SS) |
| 1430613 | 1432805 | + | *vgrG1* | Secretion system | Hcp secretion island-1 encoded type VI secretion system (H-T26SS) |
| 1418960 | 1419715 | - | *aec26* | Secretion system | ACE T6SS(*Escherichia*) |
| 1416240 | 1418951 | - | *aec27/clpV* | Secretion system | ACE T7SS(*Escherichia*) |
| 1406090 | 1406563 | - | *aec32* | Secretion system | ACE T8SS(*Escherichia*) |
| 3513907 | 3514425 | - | *bicA* | Secretion system | Bsa T3SS(*Burkholderia*) |
| 3516634 | 3517293 | - | *spaP* | Secretion system | Bsa T4SS(*Burkholderia*) |
| 2394621 | 2395034 | - | *flgB* | Secretion system | Flagella (cluster I)(*Yersinia*) |
| 2394211 | 2394615 | - | *flgC* | Secretion system | Flagella (cluster I)(*Yersinia*) |
| 2392149 | 2393369 | - | *flgE* | Secretion system | Flagella (cluster I)(*Yersinia*) |
| 2391372 | 2392127 | - | *flgF* | Secretion system | Flagella (cluster I)(*Yersinia*) |
| 2389764 | 2390507 | - | *flgH* | Secretion system | Flagella (cluster I)(*Yersinia*) |
| 2387657 | 2388643 | - | *flgJ* | Secretion system | Flagella (cluster I)(*Yersinia*) |
| 2385895 | 2387538 | - | *flgK* | Secretion system | Flagella (cluster I)(*Yersinia*) |
| 2396539 | 2396838 | + | *flgM* | Secretion system | Flagella (cluster I)(*Yersinia*) |
| 2401157 | 2402305 | - | *flhB* | Secretion system | Flagella (cluster I)(*Yersinia*) |
| 2414966 | 2415547 | - | *flhC* | Secretion system | Flagella (cluster I)(*Yersinia*) |
| 2415550 | 2415900 | - | *flhD* | Secretion system | Flagella (cluster I)(*Yersinia*) |
| 2372944 | 2373255 | - | *fliE* | Secretion system | Flagella (cluster I)(*Yersinia*) |
| 2373532 | 2375253 | + | *fliF* | Secretion system | Flagella (cluster I)(*Yersinia*) |
| 2375250 | 2376248 | + | *fliG* | Secretion system | Flagella (cluster I)(*Yersinia*) |
| 2378367 | 2378813 | + | *fliJ* | Secretion system | Flagella (cluster I)(*Yersinia*) |
| 2380403 | 2380885 | + | *fliL* | Secretion system | Flagella (cluster I)(*Yersinia*) |
| 2380891 | 2381922 | + | *fliM* | Secretion system | Flagella (cluster I)(*Yersinia*) |
| 2381915 | 2382325 | + | *fliN* | Secretion system | Flagella (cluster I)(*Yersinia*) |
| 2383560 | 2383829 | + | *fliQ* | Secretion system | Flagella (cluster I)(*Yersinia*) |
| 2383835 | 2384617 | + | *fliR* | Secretion system | Flagella (cluster I)(*Yersinia*) |
| 2368183 | 2368581 | + | *fliS* | Secretion system | Flagella (cluster I)(*Yersinia*) |
| 2362071 | 2362601 | - | *fliZ* | Secretion system | Flagella (cluster I)(*Yersinia*) |
| 1797623 | 1798378 | + |  | Secretion system | T4SS effectors(*Coxiella*) |
| 1427614 | 1428114 | - |  | Secretion system | T6SS(*Aeromonas*) |
| 1426115 | 1427593 | - |  | Secretion system | T7SS(*Aeromonas*) |
| 1430011 | 1430529 | + | *hcp* | Secretion system | T8SS(*Aeromonas*) |
| 3856836 | 3857354 | + | *hcp* | Secretion system | T8SS(*Aeromonas*) |
| 3519833 | 3521131 | - | *ysaN* | Secretion system | Ysa TTSS(*Yersinia*) |
| 3516380 | 3516628 | - | *ysaS* | Secretion system | Ysa TTSS(*Yersinia*) |
| 3521557 | 3523656 | - | *ysaV* | Secretion system | Ysa TTSS(*Yersinia*) |
| 267396 | 268769 | - | *hlyA* | Toxin | Hemolysin HlyA(*Aeromonas*) |
| 2770489 | 2772165 | + | *rtxB* | Toxin | RTX toxin(*Vibrio*) |
| 558216 | 558833 | + | *ureG* | Acid resistance | Urease(*Helicobacter*) |
| 771352 | 772542 | - |  | Efflux pump | AcrAB(*Klebsiella*) |
| 768179 | 771337 | - |  | Efflux pump | AcrAB(*Klebsiella*) |
| 463169 | 466303 | + | *acrB* | Efflux pump | AcrAB(*Klebsiella*) |
| 1071721 | 1073238 | - | *farB* | Efflux pump | FarAB(*Neisseria*) |
| 54089 | 55549 | + |  | Endotoxin | LOS(*Haemophilus*) |
| 1004383 | 1004961 | + | *gmhA/lpcA* | Endotoxin | LOS(*Haemophilus*) |
| 2433464 | 2434405 | + | *htrB* | Endotoxin | LOS(*Haemophilus*) |
| 1768955 | 1769809 | + | *kdsA* | Endotoxin | LOS(*Haemophilus*) |
| 29039 | 30316 | - | *kdtA* | Endotoxin | LOS(*Haemophilus*) |
| 3076499 | 3077302 | - | *lpxA* | Endotoxin | LOS(*Haemophilus*) |
| 307531 | 3076491 | - | *lpxB* | Endotoxin | LOS(*Haemophilus*) |
| 2826849 | 2827766 | - | *lpxC* | Endotoxin | LOS(*Haemophilus*) |
| 3077885 | 3078913 | - | *lpxD* | Endotoxin | LOS(*Haemophilus*) |
| 2949004 | 2949726 | + | *lpxH* | Endotoxin | LOS(*Haemophilus*) |
| 1388627 | 1389628 | + | *lpxK* | Endotoxin | LOS(*Haemophilus*) |
| 1386885 | 1388630 | + | *msbA* | Endotoxin | LOS(*Haemophilus*) |
| 37772 | 38728 | - | *opsX/rfaC* | Endotoxin | LOS(*Haemophilus*) |
| 989220 | 989813 | - | *orfM* | Endotoxin | LOS(*Haemophilus*) |
| 39787 | 40725 | - | *rfaD* | Endotoxin | LOS(*Haemophilus*) |
| 3179527 | 3180951 | - | *rfaE* | Endotoxin | LOS(*Haemophilus*) |
| 38725 | 39777 | - | *rfaF* | Endotoxin | LOS(*Haemophilus*) |
| 188052 | 189143 | + | *wecA* | Endotoxin | LOS(*Haemophilus*) |
| 2179521 | 2180081 | - | *fimA* | Fimbrial adherence determinants | Fim(*Salmonella*) |
| 1745868 | 1746536 | + | *pefD* | Fimbrial adherence determinants | Pef(*Salmonella*) |
| 927474 928232 | 928232 | + | *stfD* | Fimbrial adherence determinants | Stf(*Salmonella*) |
| 918187 | 918945 | + | *stfD* | Fimbrial adherence determinants | Stf(*Salmonella*) |
| 58410 | 59225 | - |  | Immune evasion | Capsule(*Acinetobacter*) |
| 2732564 | 2733580 | - | *galE* | Immune evasion | Exopolysaccharide(*Haemophilus*) |
| 2207345 | 2208250 | - | *galU* | Immune evasion | Exopolysaccharide(*Haemophilus*) |
| 4002775 | 4003737 | + | *galU* | Immune evasion | Exopolysaccharide(*Haemophilus*) |
| 295066 | 296403 | + | *mrsA/glmM* | Immune evasion | Exopolysaccharide(*Haemophilus*) |
| 3588130 | 3589776 | - | *pgi* | Immune evasion | Exopolysaccharide(*Haemophilus*) |
| 2404407 | 2405459 | - | *cheB* | Invasion | Flagella(*Burkholderia*) |
| 2405452 | 2406270 | - | *cheR* | Invasion | Flagella(*Burkholderia*) |
| 2410109 | 2410609 | - | *cheW* | Invasion | Flagella(*Burkholderia*) |
| 2403958 | 2404347 | - | *cheY* | Invasion | Flagella(*Burkholderia*) |
| 2403292 | 2403948 | - | *cheZ* | Invasion | Flagella(*Burkholderia*) |
| 2413938 | 2414831 | - | *motA* | Invasion | Flagella(*Burkholderia*) |
| 3602633 | 3603940 | - | *icl* | Lipid and fatty acid metabolism | Isocitrate lyase(*Mycobacterium*) |
| 851212 | 851592 | - | *panD* | Lipid and fatty acid metabolism | Pantothenate synthesis(*Mycobacterium*) |
| 2419224 | 2419922 | - | *mgtC* | Magnesium uptake | Mg^2+^ transport(*Salmonella*) |
| 2365239 | 2366351 | - | *flaA* | Motility | Flagella(*Bordetella*) |
| 2363826 | 2364929 | - | *flaA* | Motility | Flagella(*Bordetella*) |
| 2412886 | 2413935 | - | *motB* | Motility | Flagella(*Bordetella*) |
| 2992588 | 2993502 | + |  | Nutritional factor | Allantoin utilization(*Klebsiella*) |
| 1365160 | 1366209 | + |  | Nutritional factor | Allantoin utilization(*Klebsiella*) |
| 1257729 | 1258766 | + | *bioB* | Nutritional virulence | Biotin metabolism(*Francisella*) |
| 699526 | 701286 | - |  | Nutritional virulence | Cysteine acquisition(*Francisella*) |
| 1828636 | 1829610 | + | *msbB2* | Others | MsbB2(*Shigella*) |
| 189167 | 190225 | + |  | Others | O-antigen(*Yersinia*) |
| 2206082 | 2207089 | - |  | Serum resistance | LPS rfb locus(*Klebsiella*) |
| 193827 | 194708 | + | *rmlA* | Serum resistance | LPS rfb locus(*Klebsiella*) |
| 3945068 | 3946441 | + |  | Serum resistance and immune evastion | LPS(*Francisella*) |
| 2035931 | 2036455 | - | *sodCI* | Stress adaptation | SodCI(*Salmonella*) |

**Table S3** Annotated genes in ICE*Pmi*W2.

| start | end | strand | gene | name |
| --- | --- | --- | --- | --- |
| 3263616 | 3263867 | + |  | helix-turn-helix domain-containing protein |
| 3263923 | 3264792 | + |  | hypothetical protein |
| 3264779 | 3265441 | + |  | DUF6475 domain-containing protein |
| 3265428 | 3265976 | + |  | lytic transglycosylase domain-containing protein |
| 3265973 | 3266272 | + | *flhD* | flagellar transcriptional regulator FlhD |
| 3266269 | 3266802 | + |  | FlhC family transcriptional regulator |
| 3266860 | 3267291 | + | *eexS* | entry exclusion protein EexS |
| 3267324 | 3270893 | - | *traG* | conjugal transfer protein TraG |
| 3270897 | 3272285 | - | *traH* | conjugal transfer protein TraH |
| 3272288 | 3273232 | - |  | thioredoxin family protein |
| 3273325 | 3274029 | - |  | hypothetical protein |
| 3274119 | 3275192 | - |  | primase-helicase zinc-binding domain-containing protein |
| 3275284 | 3275625 | - |  | hypothetical protein |
| 3275625 | 3276122 | - | *radC* | DNA repair protein RadC |
| 3276205 | 3277860 | - |  | VWA domain-containing protein |
| 3277930 | 3278370 | - |  | hypothetical protein |
| 3278432 | 3279385 | - |  | DUF3150 domain-containing protein |
| 3279484 | 3280251 | - |  | hypothetical protein |
| 3280251 | 3281210 | - |  | CbbQ/NirQ/NorQ C-terminal domain-containing protein |
| 3281420 | 3282436 | - | *yqaJ* | YqaJ viral recombinase family protein |
| 3282497 | 3282640 | - |  | hypothetical protein |
| 3282723 | 3283541 | - | *bet* | phage recombination protein Bet |
| 3283621 | 3284040 | - | *ssb* | single-stranded DNA-binding protein |
| 3284056 | 3284382 | - |  | hypothetical protein |
| 3284749 | 3285351 | + |  | hypothetical protein |
| 3285581 | 3285988 | - | *cadR* | Cd(II)/Pb(II)-responsive transcriptional regulator |
| 3286084 | 3286980 | + |  | cation transporter |
| 3286984 | 3287496 | + | *lspA* | signal peptidase II |
| 3287518 | 3288807 | + |  | ISL3-like element ISPpu12 family transposase |
| 3288940 | 3289778 | - |  | AadA family aminoglycoside 3''-O-nucleotidyltransferase |
| 3289863 | 3291083 | - | *ere(A)* | EreA family erythromycin esterase |
| 3291276 | 3291749 | - | *dfrA32* | trimethoprim-resistant dihydrofolate reductase DfrA32 |
| 3291907 | 3292704 | + | *intI1* | class 1 integron integrase IntI1 |
| 3292757 | 3293461 | - | *IS26* | IS6-like element IS26 family transposase |
| 3293512 | 3293604 | - | *padR* | PadR family transcriptional regulator |
| 3293615 | 3294820 | - | *chrA* | chromate efflux transporter |
| 3294976 | 3295179 | - |  | hypothetical protein |
| 3295267 | 3295971 | + | *IS26* | IS6-like element IS26 family transposase |
| 3296165 | 3296551 | + | *bleO* | bleomycin binding protein |
| 3296871 | 3297263 | - |  | pyridoxamine 5'-phosphate oxidase family protein |
| 3297459 | 3298163 | + | *IS26* | IS6-like element IS26 family transposase |
| 3298327 | 3299166 | - | *sul1* | sulfonamide-resistant dihydropteroate synthase Sul1 |
| 3299160 | 3299507 | - | *qacE*△*l* | quaternary ammonium compound efflux SMR transporter QacE△1 |
| 3299730 | 3300182 | - | *arr-3* | NAD(+)--rifampin ADP-ribosyltransferase Arr-3 |
| 3300267 | 3300899 | - | *catB3* | type B-3 chloramphenicol O-acetyltransferase CatB3 |
| 3301037 | 3301867 | - | *bal_OXA-1_* | oxacillin-hydrolyzing class D beta-lactamase OXA-1 |
| 3302028 | 3303353 | - | *ltrA* | group II intron reverse transcriptase/maturase |
| 3303838 | 3304392 | - | *aac(6’)-Ib-cr5* | fluoroquinolone-acetylating aminoglycoside 6'-N-acetyltransferase AAC(6')-Ib-cr5 |
| 3304536 | 3305240 | - | *IS26* | IS6-like element IS26 family transposase |
| 3305396 | 3305446 | + |  | transposase |
| 3305546 | 3305746 | + |  | DUF3330 domain-containing protein |
| 3305872 | 3306432 | + |  | recombinase family protein |
| 3306435 | 3309386 | + | *TnAs3* | Tn3-like element TnAs3 family transposase |
| 3309320 | 3309796 | + |  | hypothetical protein |
| 3309880 | 3310584 | - | *IS1006* | IS6-like element IS1006 family transposase |
| 3310648 | 3311478 | + | *ISVsa3* | IS91-like element ISVsa3 family transposase |
| 3311509 | 3312393 | + |  | DUF3363 domain-containing protein |
| 3312610 | 3313824 | + | *floR* | chloramphenicol/florfenicol efflux MFS transporter FloR |
| 3313852 | 3314157 | + |  | LysR family transcriptional regulator |
| 3314269 | 3314673 | + | *IS91* | IS91 family transposase |
| 3314780 | 3315616 | - | *aph(6)-Id* | aminoglycoside O-phosphotransferase APH(6)-Id |
| 3315616 | 3316419 | - | *aph(3'')-Ib* | aminoglycoside O-phosphotransferase APH(3'')-Ib |
| 3316480 | 3317295 | - | *sul2* | sulfonamide-resistant dihydropteroate synthase Sul2 |
| 3317668 | 3318821 | + | *IS3* | IS3 family transposase |
| 3318811 | 3319002 | + |  | hypothetical protein |
| 3319074 | 3320255 | + |  | DUF4268 domain-containing protein |
| 3320412 | 3322562 | + | *mobH* | MobH family relaxase |
| 3322611 | 3324431 | + | *traD* | conjugative transfer system coupling protein TraD |
| 3324441 | 3325001 | + |  | hypothetical protein |
| 3324988 | 3325623 | + |  | DUF4400 domain-containing protein |
| 3325737 | 3326333 | + |  | hypothetical protein |
| 3326326 | 3327432 | + |  | XRE family transcriptional regulator |
| 3327569 | 3327850 | + | *traL* | type IV conjugative transfer system protein TraL |
| 3327847 | 3328473 | + | *traE* | TraE family type IV conjugative transfer system protein |
| 3328457 | 3329353 | + | *traK* | type-F conjugative transfer system secretin TraK |
| 3329356 | 3330645 | + | *traB* | TraB/VirB10 family protein |
| 3330720 | 3331292 | + | *traV* | type IV conjugative transfer system lipoprotein TraV |
| 3331289 | 3331675 | + | *traA* | TraA family conjugative transfer protein |
| 3331896 | 3332687 | + | *abiEi* | type IV toxin-antitoxin system AbiEi family antitoxin |
| 3332680 | 3333618 | + | *abiEii/abiGii* | nucleotidyl transferase AbiEii/AbiGii toxin family protein |
| 3333750 | 3334442 | + | *dsbC* | DsbC family protein |
| 3334442 | 3336841 | + | *traC* | type IV secretion system protein TraC |
| 3336834 | 3337181 | + |  | hypothetical protein |
| 3337165 | 3337677 | + | *S26* | S26 family signal peptidase |
| 3337769 | 3338812 | + | *trbC* | TrbC family F-type conjugative pilus assembly protein |
| 3338796 | 3339824 | + | *traU* | TraU family protein |
| 3339827 | 3343519 | + | *traN* | conjugal transfer protein TraN |
| 3343978 | 3345396 | + | *sir2* | SIR2 family protein |
| 3345393 | 3347432 | + |  | DUF87 domain-containing protein |
| 3347841 | 3348515 | - |  | endonuclease |
| 3348557 | 3349844 | - | *ISL3* | ISL3 family transposase |
| 3349866 | 3350378 | - | *lspA* | signal peptidase II |
| 3350382 | 3351278 | - |  | cation transporter |
| 3351374 | 3351781 | + | *cadR* | Cd(II)/Pb(II)-responsive transcriptional regulator |
| 3352236 | 3352514 | - |  | tyrosine-type recombinase/integrase |
| 3352526 | 3352717 | - |  | hypothetical protein |
| 3352722 | 3353060 | - |  | hypothetical protein |
| 3352964 | 3354154 | - | *tet(C)* | tetracycline efflux MFS transporter Tet(C) |
| 3354247 | 3354906 | + | *tetR(C)* | tetracycline resistance transcriptional repressor TetR(C) |
| 3355273 | 3355425 | - |  | hypothetical protein |
| 3355739 | 3356443 | + | *IS6* | IS6-like element IS26 family transposase |
| 3356633 | 3357448 | - | *aph(3’)-Ia* | aminoglycoside O-phosphotransferase APH(3')-Ia |
| 3357578 | 3358282 | + | *IS26* | IS6-like element IS26 family transposase |
| 3358336 | 3358938 | - | *IS5* | IS5 family transposase |
| 3358973 | 3359848 | - | *bla_CTX-M-65_* | extended-spectrum class A beta-lactamase CTX-M-65 |
| 3360098 | 3360289 | - | *IS1380* | IS1380 family transposase |
| 3360353 | 3360481 | - |  | hypothetical protein |
| 3360979 | 3361485 | - |  | hypothetical protein |
| 3361577 | 3362281 | - | *IS26* | IS6-like element IS26 family transposase |
| 3362667 | 3363083 | + | *fosA3* | fosfomycin resistance glutathione transferase FosA3 |
| 3363088 | 3363606 | - |  | FidL-like protein |
| 3363606 | 3364394 | - |  | winged helix-turn-helix domain-containing protein |
| 3364414 | 3364839 | - | *tetR/acrR* | TetR/AcrR family transcriptional regulator |
| 3364894 | 3365598 | - | *IS26* | IS6-like element IS26 family transposase |
| 3365712 | 3366488 | + | *aac(3)-IVa* | aminoglycoside N-acetyltransferase AAC(3)-IVa |
| 3366717 | 3367742 | + | *aph(4)-Ia* | aminoglycoside O-phosphotransferase APH(4)-Ia |
| 3368164 | 3368916 | - |  | IS6 family transposase |
| 3368978 | 3370501 | + |  | Tn3 family transposase |
| 3370561 | 3370689 | + |  | hypothetical protein |
| 3370727 | 3371212 | + |  | phenol hydroxylase |
| 3371409 | 3372499 | - |  | IS4-like element ISAba1 family transposase |
| 3372589 | 3373403 | + | *sul2* | sulfonamide-resistant dihydropteroate synthase Sul2 |
| 3373490 | 3373792 | + |  | phosphoglucosamine mutase |
| 3373968 | 3375461 | - |  | IS91-like element ISVsa3 family transposase |
| 3375879 | 3378857 | - |  | Tn3 family transposase |
| 3378971 | 3379564 | - |  | Tn3 family transposase |
| 3379917 | 3381020 | + | *umuC* | translesion error-prone DNA polymerase V subunit UmuC |
| 3381031 | 3381474 | - | *mobI* | conjugative transfer protein MobI(A/C) |
| 3381939 | 3382913 | + |  | ParM/StbA family protein |
| 3383039 | 3383185 | + |  | hypothetical protein |
| 3383187 | 3384428 | + |  | integrase family protein |
| 3384445 | 3384639 | - |  | DNA-binding protein |
| 3385221 | 3385313 | - |  | peptide chain release factor 3 |
| 3385592 | 3385723 | - |  | peptidase S24 |
| 3386101 | 3387015 | - | *abiEii/abiGii* | nucleotidyl transferase AbiEii/AbiGii toxin family protein |
